# Gonadal miRNomes and transcriptomes in infected fish reveal sexually dimorphic patterns of the immune response

**DOI:** 10.1007/s10142-025-01537-w

**Published:** 2025-01-30

**Authors:** Tosca A. van Gelderen, Pinky Debnath, Silvia Joly, Edgar Bertomeu, Neil Duncan, Dolors Furones, Laia Ribas

**Affiliations:** 1 Institut de Ciències del Mar, Consejo Superior de Investigaciones Científicas (ICM-CSIC), Barcelona, 08003 Spain; 2 Department of Fish Biology and Genetics, Sylhet Agricultural University, Sylhet, 3100 Bangladesh; 3 Institut de Recerca i Tecnologia Agroalimentaries (IRTA), La Ràpita, Aquaculture, Spain

**Keywords:** Aquaculture, Infection, Marker, Immune, Reproduction, Post-transcription *Dicentrarchus labrax*, *Vibrio anguillarum*

## Abstract

Fish disease outbreaks caused by bacterial burdens are responsible for decreasing productivity in aquaculture. Unraveling the molecular mechanisms activated in the gonads after infections is pivotal for enhancing husbandry techniques in fish farms, ensuring disease management, and selecting the most resilience phenotype. The present study, with an important commercial species the European sea bass (*Dicentrarchus labrax*), an important commercial species in Europe, examined changes in the miRNome and transcriptome 48 h after an intraperitoneal infection with *Vibrio anguillarum*. The findings indicate that following infection, testes exhibited more pronounced alterations in both the miRNome and transcriptome. Specifically, males showed approximately 26% more differentially expressed genes in testicular genes compared to females (2,624 vs. 101 DEGs). Additionally, four miRNAs (miR-183-5p, miR-191-3p, miR-451-5p, and miR-724-5p) were significantly expressed post-infection in males, while none were identified in females. Interestingly, upon deep analysis of sexual dimorphic gene modules, a larger number of miRNAs were identified in infected females targeting genes related to the immune system compared to infected males. These results suggest that fish ovaries demonstrate greater resilience in response to infections by suppressing genes related to the immune system through a post-transcriptional mechanism performed by miRNAs. In contrast, testes activate genes related to the immune system and repress genes related to cellular processes to cope with the infection. In particular, the crosstalk between the miRNome and transcriptome in infected males revealed a pivotal gene, namely, insulin-like growth factor binding protein (*igfbp*), acting as a gene network hub in which miR-192-3p was connected. The current study elucidated the need to comprehend the basic immune regulatory responses associated with miRNAs and gene regulation networks that depend on fish sex. The data reveal the importance of considering sex as a factor in interpreting the immune system in fish to generate efficient protocols to prevent outbreaks in fish farms.

**Supplementary Information:**

The online version contains supplementary material available at 10.1007/s10142-025-01537-w.

## Abstract (plain language summary)

This study investigates how European sea bass (*Dicentrarchus labrax*) respond to infections caused by the bacteria *Vibrio anguillarum*, with a focus on understanding the molecular mechanisms in the gonads. Fish disease outbreaks are a major issue in aquaculture, and knowing how fish resist infections can improve disease management. Researchers analyzed changes in the miRNome (microRNAs) and transcriptome in male and female European sea bass 48 h after infection. The results showed that males had more significant changes in gene expression compared to females, with 2,624 differentially expressed genes in testes vs. 101 in ovaries. Four specific miRNAs were active in males post-infection, but none were found in females. However, females exhibited a higher number of miRNAs targeting immune-related genes, suggesting that ovaries may suppress immune responses better than testes. Males, on the other hand, activated immune-related genes while suppressing cellular processes to fight the infection. A key gene, insulin-like growth factor binding protein (*igfbp*), was identified as important in male immune response, connected to miR-192-3p. This study highlights the importance of considering fish sex as a relevant factor when studying immune responses, as it could help develop better disease prevention strategies in aquaculture.

**Supplementary Information:**

The online version contains supplementary material available at 10.1007/s10142-025-01537-w.

## Highlights

- Testes showed more molecular changes at the microRNome and transcriptome levels in response to infection than ovaries.
- In testes of infected fish, miR-183-5p and miR-191-3p were upregulated while miR-451-5p and miR-724-5p were downregulated.
- Larger number of target genes were inhibited in females which were mostly related to the immune system.
- Importance of considering sexual dimorphism in immune responses was highlighted.

**Supplementary Information:**

The online version contains supplementary material available at 10.1007/s10142-025-01537-w.

## Background

Aquaculture is a crucial industry for fish supply around the world. To achieve high production rates, fish could be subjected to a variety of stressful conditions, including high stocking density, poor water quality, and inadequate nutrition, all of which have the potential to cause the emergence of pathogenic diseases. Disease outbreaks are a frequent problem in aquaculture and affect the welfare as well as nutritional and commercial yield, among others (FAO 2022). A common pathogen in aquaculture is *Vibrio anguillarum*, a Gram-negative bacterium that causes vibriosis in crustaceans, bivalves and fish, inducing symptoms such as haemorrhages, ulceration and necrosis, appetite loss among others (Frans et al. 2011; Hickey and Lee 2018). This pathogen is responsible for significant losses in aquaculture production worldwide being one of the most common aquacultural diseases in Asia (Ina-Salwany et al. 2019), but also affecting Mediterranean aquaculture farms (Muniesa et al. 2022). In particularly, European sea bass (*Dicentrachus labrax*) together with other important commercial species in Europe such as the gilthead sea bream (*Sparus aurata*), are threatened by these bacterial infections (Muniesa et al. 2020).

Micro RNAs (miRNAs) are short (17–22 nucleotides) non-coding RNAs that suppress protein synthesis post-transcriptionally by binding to complementary regions in their target mRNA sequence (Ambros 2004). Since their initial detection, miRNAs have served as valuable instruments in a wide array of applications in human health diagnostics, particularly in tumor identification in cancer (Hamam et al. 2017). The notable characteristic that makes miRNAs valuable markers for long-lasting molecular processes is their substantial preservation of both structure and function across evolutionary time (Niwa and Slack 2007), paving the way for their discovery in many species. In fish, following the initial discovery of miRNAs in zebrafish (Lim et al. 2003), numerous investigations have been conducted to characterize the miRNA repertoire in other teleost species. In aquaculture, the collection of miRNAs, along with the currently described patterns of miRNA expression, has placed miRNAs at the forefront of promising indicators for enhancing productivity. These indicators include refining selection for breeding programs. For example, recently in rainbow trout (*Onchorynchus mykiss*), in which circulating miRNAs in the bloodstream were recognized as a non-invasive method to elucidate metabolic and reproductive conditions (Cardona et al. 2021).

The miRNAs are associated with numerous biological processes, such as cell division and proliferation, embryonic growth, apoptosis, metabolism, immunity, and gametogenesis, among others (Niwa and Slack 2007; Vidigal and Ventura 2015; Avital et al. 2018). In mammals, miRNAs play significant roles during infection by a variety of pathogens, such as viruses, parasites, and bacteria (Eulalio et al. 2012; Staedel and Darfeuille 2013). Similarly in teleosts, numerous studies exposed the role of miRNAs in both innate and adaptive immune responses. To date, many studies documented the contribution of miRNAs to pathogenic bacteria responses in commercial fish species, for example: *V. anguillarum* in turbot (*Scophthalmus maximus*) (Gao et al. 2019a), *Streptococcus agalactiae* in Nile tilapia (*Oreochromis niloticus*) (Gao et al. 2019b), bacterial and virus infections in zebrafish (*Danio rerio*) (Ji et al. 2019), *Staphylococcus aureus* in zebrafish adults (Zhang et al. 2019), and *Aeromonas salmonicida* subsp. *salmonicida* in rainbow trout (Cao et al. 2019), and more recently *Aeromonas hydrophila* in grass carp kidney cells (Li et al. 2024). In Atlantic salmon (*Salmon salar*) macrophages infected with *A. salmonicida*, revealed the alteration of three miRNAs (miR-21-5p, 223-3p, and 223-5p) with which functions that appear to be connected to pathogenesis, stress response, and allosteric exchange of histones (Hairul-Islam et al. 2014). Additionally, miR-21-5p, miR-202-5p, miR-451, miR-30a-5p contributed to versatile functions, including immune regulation in teleosts across evolution (Qiu et al. 2018; Liyanage et al. 2020).

Fish reproduction involves complex processes regulated by numerous molecules and signaling networks at the transcriptional and post-transcriptional level in which miRNAs play significant role in gonadal development in fish (Alvi et al. 2021; Bhat et al. 2021). Recent research, has found numerous sex-biased miRNAs in aquaculture species: spotted knifejaw (*Oplegnathus punctatus*) (Du et al. 2018), sturgeon (*Acipenser schrenckii*) (Zhang et al. 2018), silver fish (*Trachinotus ovatus*) (He et al. 2019), *Odontobutis potamophila* (Zhao et al. 2017) and European sea bass (Sarropoulou et al. 2019). For example, miR-21-5p, and miR-462-5p were highly expressed in the ovary in yellow catfish (*Pelteobagrus fulvidraco*) (Jing et al. 2014), whereas others (e.g., miR-9-3p, miR-103b-3p, and miR-7b) were primarily expressed in the testis (Wang et al. 2018). Meanwhile, several miRNAs, including miR-29a, miR-34 or miR-143, showed tissue specificity and sexually dimorphic expression patterns in Atlantic halibut (*Hippoglossus hippoglossus*) (Bizuayehu et al. 2012). These investigations clarify the reproduction-related roles played by miRNAs in the gonadal tissue in fish. However, to date, we understand that no research has investigated how miRNAs regulate the gonadal immune function during infections.

Exploring the interaction between the immune and reproductive systems holds significant value. It not only aids in controlling pathogen transmission in fish farms but also sheds light on how fish combat infections in their reproductive organs. These organs enjoy immune privilege, shielding germ cells responsible for passing on parental genetic material from pathogens. Nevertheless, sometimes, infections in the gonads result in transferring either the pathogen, for example that described in zebrafish after parasitic infection (Caballero-Huertas et al. 2021), viral infections in Gilthead sea bream (*Sparus aurata*) or European sea bass (Padrós et al. 2022) or transferring immune-epigenetic memory (Beemelmanns and Roth 2017; Pierron et al. 2021). Knowledge of the miRNA plethora and their post-transcriptional regulatory mechanisms in the infected gonads may advance our understanding of host-pathogen interactions. Sex is a crucial factor influencing the immune response in vertebrates, including fish (Caballero-Huertas et al., 2024). Fish infection prevalence by sex refers to the varying rates and outcomes of infections in males and females. Thus, it can be hypothesized that the immune response in the gonads will differ between sexes and the understanding the differences in susceptibility, severity, and frequency of infections based on sex will help to development immunological strategies for disease management and prevention in cultured fish and to prevent vertical transmission from broodstock to offspring. The purpose of the current study was to investigate the alterations of the miRNome and the transcriptome in the gonads of a relevant commercial Mediterranean species, the European sea bass, subjected to a bacterial infection commonly found in fish farms.

## Methods

### Experimental fish

Unvaccinated European sea bass fry (3.9 g) were obtained from a commercial Spanish hatcheryand raised during three years at the Institute of Agrifood Research and Technology (IRTA, Tarragona, Spain), to provide a broodstock of 32 months of age. Animals (N = 104) fish with mean weight of 663 ± 162 g were chipped and sexed. Throughout the period of rearing, commercial feed (Skretting) was provided to all the fish in accordance with feeding tables. Fish were held in tanks (2,000–10,000 L) connected to an IRTAmar^®^ water recirculation aquaculture system (RAS), where the fish were maintained in flowthrough or RAS mode until the challenge experiment. Photoperiod was natural from natural daylight. The water temperature was natural (10–22 °C). Temperature and dissolved oxygen (6.4 ± 0.6 mg L − 1; OXI330, Crison Instruments) were measured daily, and pH (7.4 ± 0.2; pHmeter 507, Crison Instruments), salinity (36‰; MASTER-20 T, ATAGO Co. Ltd), ammonia (0.14 ± 0.1 mg NH4 + L − 1) and nitrite (0.2 ± 0.1 mg NO2 − L−1) levels (HACH DR9000 Colorimeter, Hach, Spain) were monitored weekly. In February, six weeks before the challenge, the breeders were sexed and individually marked for identification with a PIT (passive integrated transponder) tag (TROVAN, Madrid, Spain).

Vibrio anguillarum *infection and sample collection*

To conduct the bacterial challenge experiment, 40 healthy fish (20 per sex) were chosen randomly from the stock. On the 5th April, the fish were transferred to IRTA’s biosafety challenge room, into two cylindrical tanks (2000 L) connected to a RAS unit (IRTAmar^®^) equipped with real-time control of oxygen and temperature, mechanical filtration, biofiltration, and ultraviolet disinfection of the water. The outflow water was chlorinated, followed by ozone treatment before being discharged. The photoperiod was natural and water quality conditions in terms of temperature and salinity were 14.1 ± 1.1 °C and 32.3 ± 0.4 ppt, respectively. Fish were given 100 ppm tricaine methane-sulfonate (MS-222, Sigma-Aldrich, Madrid, Spain) anesthesia before being intraperitoneally (IP) injected with sublethal dose (10E + 05 CFU/fish) of *V. anguillarum*. Phosphate Buffer Saline (PBS) was IP injected as a control. Fish were divided into two tanks, one for infected animals (ten males and ten females) and one for uninfected animals (ten males and ten females). Daily observations of fish were made to monitor the wellbeing of the fish. Over the duration of the experiment, no mortalities were found. After 48 h, fish were euthanized under an overdose of MS-222 and assessed for biometry (standard length and weight). For each gonadal tissue, the gonads were cut into three pieces, two for sequencing analysis which were flash frozen in liquid nitrogen and kept at -80°C, and the other one for histological purposes. Further, visual assessment of the gonadal maturation was identified to indicate the maturation stages of the gonads (Carrillo et al. 2015).

### Microbiological postmortem analyses

Microbiological postmortem sampling of head kidney and gonad was conducted to assess *V. anguillarum* presence in both tissue compartments. Sterile swaps were soaked in each of the tissue of freshly dead fish, and plated onto general media TSA- NaCl (Trypto– Casein Soy Agar, Difco laboratories) supplemented with 0.5% of NaCl) and onto Vibrionaceae selective media TCBS (Thiosulfate-Citrate-Bile Salts-Sucrose, DIFCO laboratories, also supplemented with NaCl) to a final concentration of 1% (w/v) agar plates, to asses viable bacterial recovery. Plates were incubated for 96 h at 23°C and were checked every 24 h for growth. Bacterial growth was recovered from the plates for DNA extraction. Additionally, PCR to determine the presence of V. *anguillarum* (Hong et al. 2007) was performed on DNA extracted by following the manufacturer’s instructions (Dneasy Blood & Tissue Kit, QIAGEN) from both tissue matrix (gonad and head kidney) and bacterial growth from plates of the same samples.

### Histological analysis

For histological study, ovaries and testes of all sampled fish (N = 40) were collected and fixed for 24 h at room temperature in 4% paraformaldehyde (PAF) in PBS, pH 7.4. After incubations, PAF was washed for 24 h in PBS and dehydrated in an ascending sequence of ethanol concentrations. Finally, tissues were cleaned in xylene, and then embedded in paraffin. Section (7 μm thick) were cut using a microtome (Reichert-Jung 2040), float mounted on glass slides, stained with haematoxylin-eosin, and mounted with DPX. Photographs were taken under a microscope objective with a magnification of 10x for testis and, of 4X for ovaries with the stereoscope (Leica M205 C).

### RNA and miRNA gonadal extraction

Total RNA from four fish per group and sex (total of 16), was isolated from gonadal samples by using an RNA isolation kit (RNeasy mini kit, Qiagen) according to the manufacturer’s instructions. Quality of the samples was assessed by Nanodrop (Agilent Technologies) with ratio 260/280 = 2.11 ± 0.05 and 260/230 = 1.89 ± 0.51) and by the RNA Integrative Number (RIN) measured with a Bioanalyzer (Agilent Technologies, USA) with values > 8.3 for testes. In ovary, RIN numbers were not considered due to naturally high levels of 5s rRNA and tRNA (Mazabraud et al. 1975; Bir et al. 2023). Nevertheless, integrity of the 18 S and 28 S was determined by the Bioanalyzer.

### Library preparations and sequencing

Libraries were prepared at BGI genomics in Hong Kong for DNBSEQ Eukaryotic Strand-specific mRNA sequencing. DNBseq with paired-end mode and read length 100 bp was performed. Filtering was done using SOAPNuke software by BGI Genomics (Chen et al. 2018). For small RNA sequencing, 16 libraries were constructed from European sea bass gonad samples. Library preparation was performed by NEBNext^®^ Small RNA Library Prep Set for Illumina^®^ (Multiplex Compatible) kit following manufacture instructions, using sequencing Lane (1 × 50, 178 v4, HiSeq) single-end mode with a read length of 50 bp at a BGI. After sequencing, data filtering was performed to removed adaptor sequences and low-quality reads from raw reads. Resulted reads were aligned to the *D. labrax* genome (version dlabrax2021) from ENSEMBL using Hisat2 (Kim et al. 2019) and counted using Featurecounts (Liao et al. 2014). The miRNAs were aligned using Prost! (Desvignes et al. 2019) to the *D. labrax* genome and annotated using medaka (*Oryzias latipes*) genome GTF file.

### Statistical analysis

Mean weight and size of the fish was calculated using R software. Data was expressed as mean ± Standard Deviation (SD) and the differences were considered significant when *p* < 0.05. Homogeneity and normality of data were analyzed by Levene test and Shapiro-Wilk normality test, respectively. Differences between control and infected groups were done by non-parametric Kruskal Wallis test. All the graphs were generated by using the ggplot2 package (v. 4.1.3) in R Studio (v. 4.1.3).

### Bioinformatics and statistical analysis

All bioinformatic analyses were done with R (R Core team 2023). Raw reads of RNA and miRNA sequencing were filtered by retaining genes and miRNAs with at least ten reads in at least four samples. In order to visualize similarities in miRNA and transcript expression patterns between samples, principal component analyses (PCA) were performed using the plotPCA function of the DESeq2 package (version 1.12.3) (Love et al. 2014).

Differential expression analysis was done using the DESeqDataSetFromMatrix, DESeq and results functions of the DESeq2 package, upholding a criterion of adjusted *p*-value < 0.05. For the differential expression analysis the FDR/adjusted p-value was determined using the Benjamin-Hochberg correction and an adjusted p value < 0.05 was upheld. Differential expression (DE) was visualized with heatmaps using the Pheatmap package (version 1.0.12). For annotation, gene names, Gene Ontology (GO) terms and GO slim terms were assigned to genes using the getBM function of the biomaRt package (version 3.18) (Durinck et al. 2005, 2009). Enriched GO terms were identified using the enricher function of the clusterProfiler package (version 3.0.4) (Yu et al. 2012; Wu et al. 2021) and GO terms with an adjusted *p*-value < 0.05 were considered significantly enriched. GO slim terms of miRNA targets were identified using BiomaRt and plotted in circular plots using the Circlize package (version 0.4.15) (Gu et al. 2014). Gene names missing from the ENSEMBL database were supplemented *via* the NCBI gene annotation database using BLAST+ (Camacho et al. 2009). Raw sequencing data generated during the current study can be found in NCBI SRA repository with the accession numbers: PRJNA1213494 and PRJNA1214368 for miRNA-seq and RNA-seq, respectively.

### Weighted correlation network analysis

Weighted Correlation Network Analysis (WGCNA) analysis was done using the method proposed by Sánchez-Baizán et al. (2022) (Sánchez-Baizán et al. 2022). Firstly, raw reads were filtered using the GoodSamplesGenes function of the WGCNA package (Langfelder and Horvath 2008, 2012). Then, reads were normalized using the *dds* function of the DESeq2 package and filtered by retaining genes and miRNAs with at least ten reads in at least four samples. The power was chosen using the PickSoftThreshold function of the WGCNA package with a scale free topology fit signed R^2^ > 0.85. Subsequently, BlockwiseModules function was used to create a Topology Overlap Matrix (TOM) and the modules were plotted in a dendrogram using the PlotDendroAndColors function. The “modules to trait correlations” were performed of the following conditions: (1) male control vs. male infected (M inf), (2) female control vs. female infected (F inf) and (3) male control vs. female control (MvF ctrl) and (4) male infected to male control (M inf). Further studies of sexual dimorphism, the comparison of male infected vs. female infected (MvF inf) was also considered.

For correlation, Pearson analysis was used (Freedman et al. 2007) and significance was calculated by Student’s *t*-test (Mishra et al. 2019). Hub genes per module were identified with the option TopHubInEachModule function. To visualize the “module membership to gene significance”, firstly the Pearson’s correlation and significance by Student’s *t* -test between the normalized reads and the module eigengenes were calculated, and, secondly, between the normalized reads and the conditions (1), (2) or (3). Finally, each module to each condition was plotted in a scatterplot using the function VerboseScatterplot, following the method proposed in Sánchez-Baizán et al. (2022).

Individual module gene networks were constructed by recalculating the TOM values and filtering by only keeping previously identified differentially expressed genes (DEGs) and differentially expressed miRNAs (DEMs) for each condition. Then, genes without annotation were removed. Networks were constructed using the function Graph_from_adjacency_matrix from the Igraph package (Csárdi et al. 2023). Subsequently, in silico targets of chosen miRNAs were identified by predicting targets and calculating context scores using the Perl script from Targetscan (version 6.0).

## Results

### Biometry

The mean weight of the female fish for the infected and control groups was 709.04 g ± 73.80 g and 698.40 g ± 69.50 g, respectively, whereas the mean weights of the male fish for the infected and control groups was 683.93 g ± 36.66 g, and 677.06 g ± 35.00 g, respectively (Supplementary Figure S1A). Infected and control fish had mean lengths of 34.05 cm ± 1.21 cm and 34.05 cm ± 1.04 cm for female. The infected male had a mean length of 33.65 cm ± 0.47 cm while the control were of 34.05 cm ± 0.72 cm (Supplementary Figure S1B). There were no significant differences in the mean weight and size of the male and female fish in the treatment and control groups, indicating that the findings were due to the treatment and not due to biometry differences among groups.

### Postmortem results

None of the tissue samples directly tested by PCR were positive for *V. anguillarum*. However, bacterial growth was recorded, albeit at very low levels, ranging from one to four colonies per plate. Specifically, bacterial growth was observed in only two animals (one female and one male) from the control group, but the PCR results for these samples were negative. In infected animals, *V. anguillarum* was detected in three testes and two ovaries, while in the head kidney, the pathogen was found in samples from one male and one female. Only one male tested positive in both tissues. The recovery of viable bacteria from the infected animals confirmed that our experimental design successfully established infection. *V. anguillarum* reached both the gonadal and head kidney tissues, yet the fish demonstrated an ability to cope with the pathogen.

### Histological analysis

In ovaries, four stages of oocyte maturation were identified: (I) primary growth stage, (II) cortical alveolus stage, (III) early-vitellogenic stage and (IV) late-vitellogenic stage. All the fish ovaries were of similar maturation independently of the treatment (Supplementary Figure S2A, B). In testes, maturation was determined based on the presence or absence of spermatozoids. All male fish, independently of the treatment, were mature as presence of spermatozoids were found inside the seminiferous tubules (Supplementary Figure S2C, D).

### RNA sequencing data

After filtering, sequencing yielded an average of 34 million reads per sample. All samples had a QC20 of > 98% and a GC count around 49%. The minimum and maximum alignment between all the sequenced samples was 93.9% and 98.3% which was aligned to the European sea bass genome and mapping with Featurecounts (Liao et al. 2014). The resulted alignment ranged in all samples between 57.3 and 68.6%. In total, 24,969 genes were identified of which 11,915 were named using ENSEMBL biomaRt (Durinck et al. 2005). Furthermore, blasting coding DNA sequences (CDS) to the NCBI *D. labrax* database yielded 16,445 named genes, with 4,953 genes not annotated by ENSEMBL biomaRt. Thus, in total, 16,868 genes were named.

### Transcriptomic alterations in the gonads after infection

**Fig. 1:**
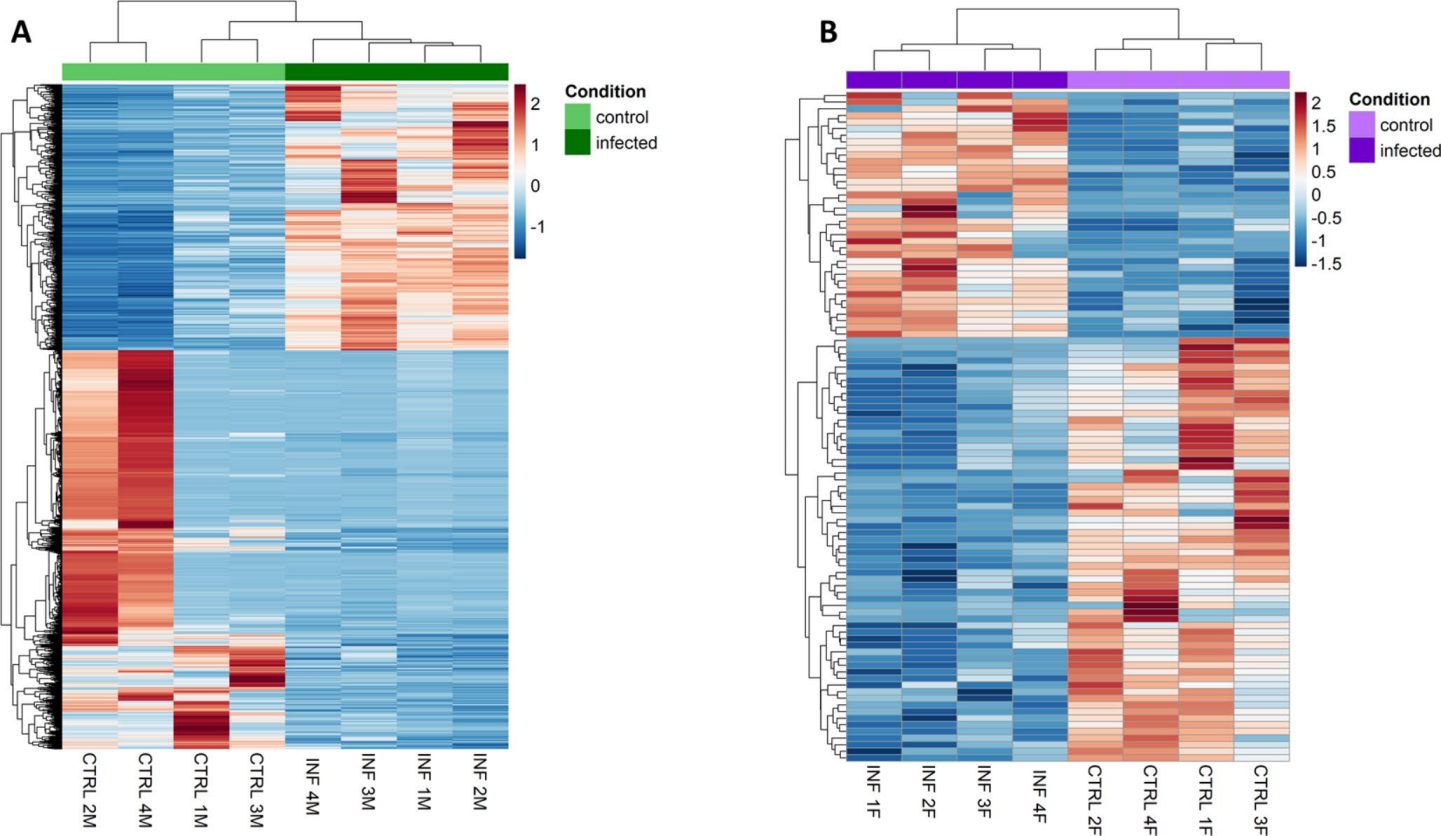
Heatmap analysis of differential expression genes obtained by RNA-sequencing of the testes (**A**) and ovaries (**B**) in European sea bass (*Dicentrarchus labrax*) 48 h after intraperitoneal infected with *Vibrio anguillarum*. A total of eight individuals per sex was used

PCA analysis clustered the samples in two groups in males, one for control and the other for infected explaining the 82% of the variance for PC1 and 10% for PC2 (Supplementary Figure S3A) while in females, PC1 explained 28% and PC2 24% (Supplementary Figure S3B). DE analysis of infected testes compared to control testes resulted in 2,624 DEGs, among which 1,050 were upregulated and 1,574 were downregulated genes (Fig. 1A, Dataset 1). In ovaries, 101 DEGs were identified, among which 37 were upregulated and 64 were downregulated (Fig. 1B, Dataset 1).

**Fig. 2:**
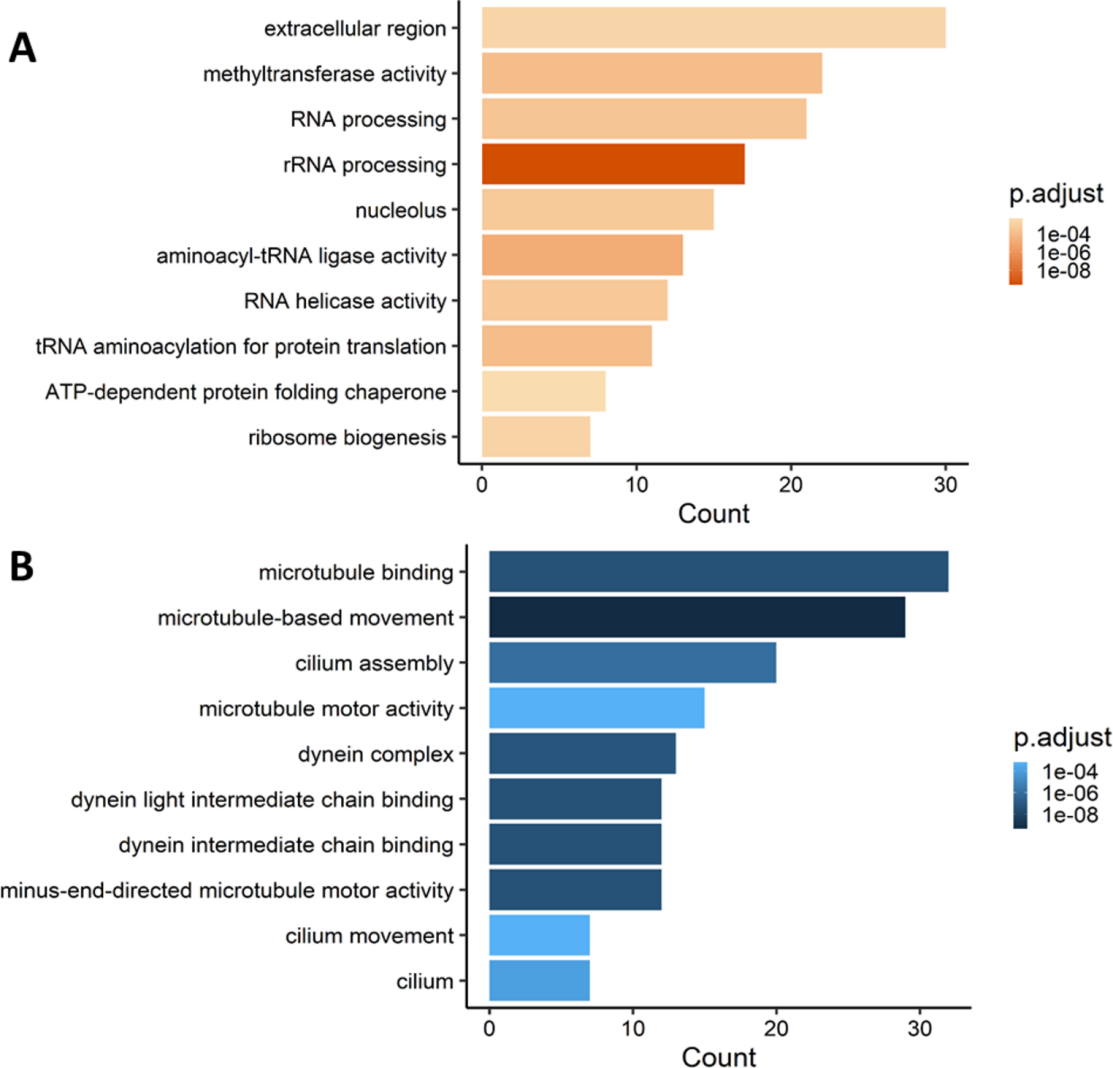
Gene Ontology terms upregulated (**A**) and downregulated (**B**) by RNA-sequencing in testes of European sea bass (*Dicentrarchus labrax*) 48 h after intraperitoneal infected with *Vibrio anguillarum*. A total of four individuals per group was used

The number of GO terms categories in the upregulated genes were: 241, 238 and 164 for Biological processes (BP), Cellular component (CC) and Molecular functions (MF), respectively. GO term enrichment analysis of testicular upregulated genes identified genes mainly related to RNA synthesis processes, such as “RNA processing”, “RNA helicase activity” and being “extracellular region” and “methyltransferase activity” with more enrichment (Fig. 2A, Dataset 2). Notably, “immune response” was also identified as an enriched GO term. The number of GO terms categories in the downregulated genes were: 181, 227 and 165 for BP, CC and MF, respectively. Testicular downregulated genes pertained mainly to microtubule activity, being “microtubule binding” and “microtubule-based movement” the two top enriched GO terms together with “cilium assembly” or “dynein complex” (Fig. 2B). No enriched GO terms were identified in ovarian DEGs.

### MicroRNome modifications in the gonads after infection

After filtering, UMI small RNA library DNBseq, resulted in an average of 25 million reads per sample with an average read length of 26 bp. A total of 212 mature miRNAs were identified (Dataset 3). PCA analysis clustered the samples in two groups in males, one for control and the other for infected, explaining 33% of the variance for PC1 and 19% for PC2 (Supplementary Figure S3C). Similarly, in females, two clusters were observed for each control and infected fish, explaining the 24% and 32% of the variance for the PC1 and PC2, respectively (Supplementary Figure S3D). In testes, four miRNAs were found to be DE in infected males two days after the infection with *V. anguillarum*, namely miR-183-5p, miR-191-3p, miR-451-5p and miR-724-5p (Dataset 4). In ovaries, no DEMs were found.

### Identification of miRNA targets in the infected testes

**Fig. 3:**
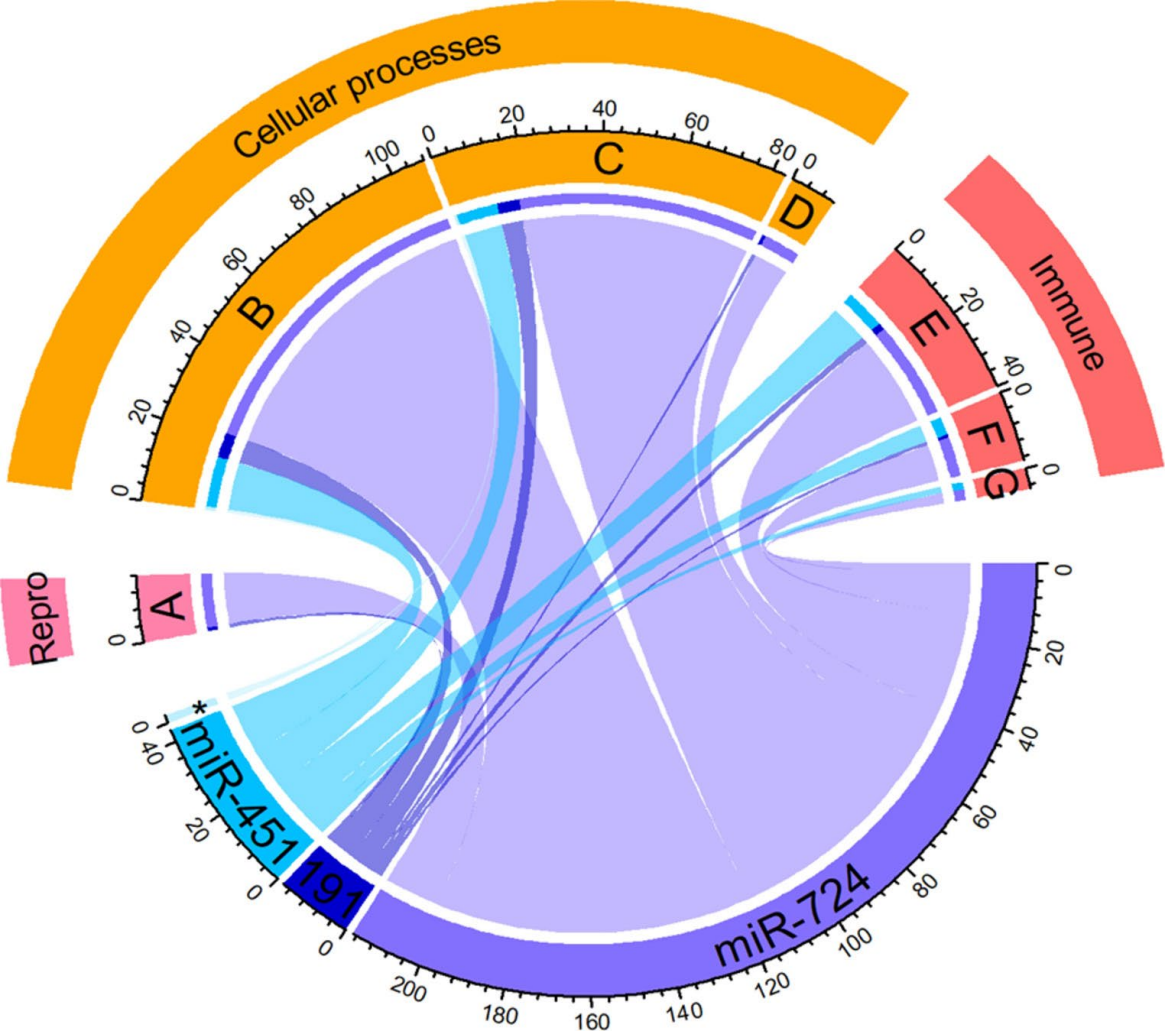
Selection of Gene Ontology terms of predicted target-genes of four miRNAs, 183-5p, miR-191-3p, miR-451-5p and miR-724-5p, found differentially expressed in the testes of European sea bass (*Dicentrarchus labrax*) 48 h after intraperitoneal infected with *Vibrio anguillarum*. Abbreviations: (**A)** transcription regulator activity, (**B)** reproductive process, (**C)** cell differentiation, (**D)** cilium organization, (**E**) immune system process, (**F**) inflammatory response, (**G**) defense response to other organism

The identified four DEMs in infected testes were further inspected by identifying potential targets. Targets were obtained by (1) negative correlation of miRNA-transcript expression by Pearson’s analysis with a *p*-value of < 0.05, and (2) in silico binding as determined by Targetscan. There were 1,693; 79; 28 and 5 potential target genes that comply with the aforementioned criteria for miR-724, miR-191, miR-451 and miR-183, respectively (Dataset 5). In total, 128 GO slim terms were assigned to the target genes, of which most genes pertained to “catalytic activity”, “organelle”, “nucleus”, “hydrolase activity”, and “transferase activity” (Dataset 6). As shown in Fig. 3, an interesting large number of potential targets of miR-451, miR-191 and miR-724 were related to the immune system by the GO terms: “immune system process”, “defense response to other organisms” and “inflammatory response”. Furthermore, some GO related to the reproduction system were identified: “reproductive process” and “cillium organization”, and to cell division: “cell differentiation process” and “transcription regulator activity”.

### Crosstalk between miRNA and genes altered after infections in the gonads

**Fig. 4:**
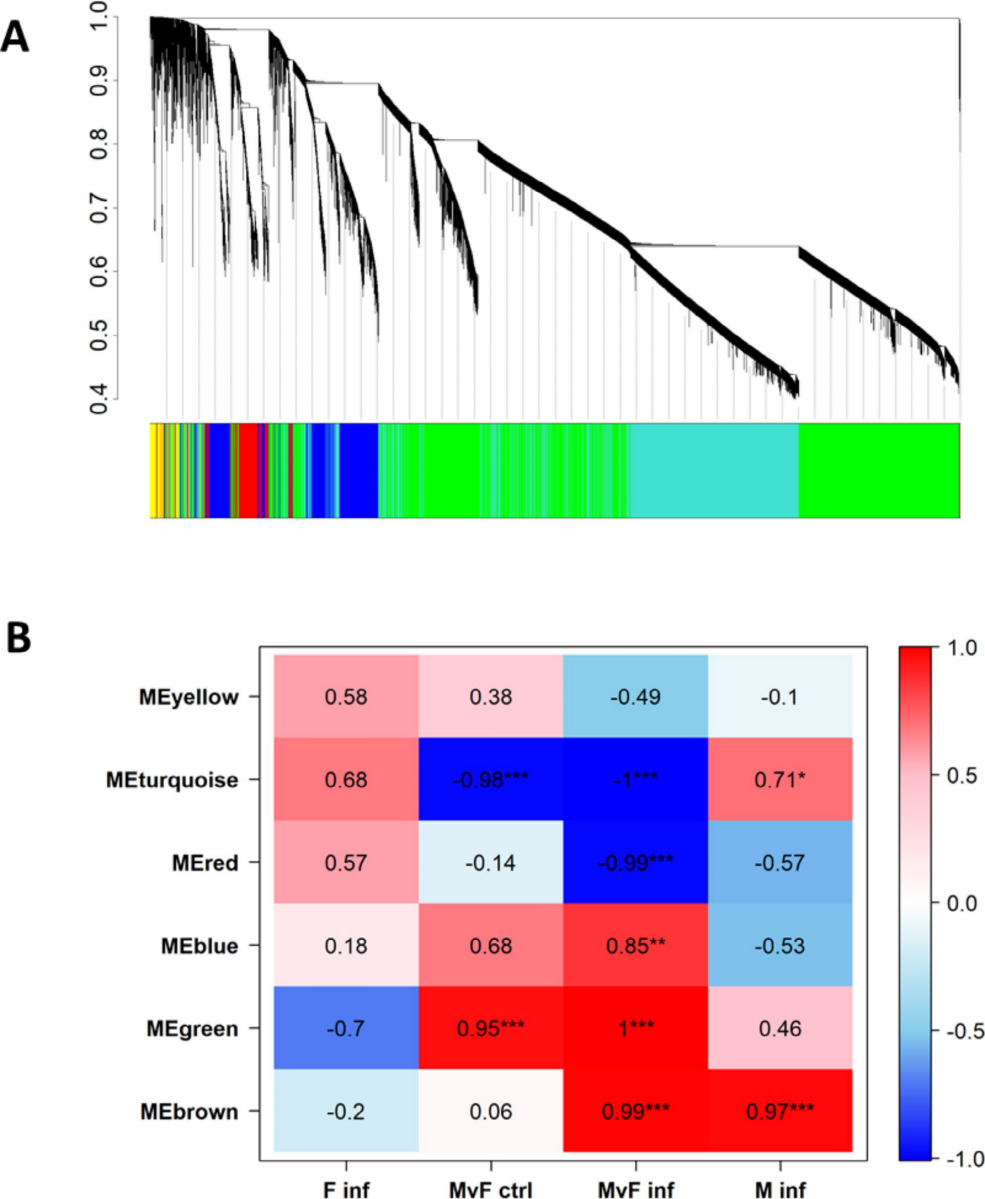
Identification of gene modules associated with sex and infection obtained from Weighted Correlation Network Analysis (WGCNA). (**A**) Gene hierarchical cluster analysis using the sea bass gonadal transcriptome obtained after RNA-sequencing in testes of European sea bass (*Dicentrarchus labrax*) 48 h after intraperitoneal infected with *Vibrio anguillarum*. (**B**) Heat map of the correlation of sex and infection with each module. Modules were correlated using a Pearson’s correlation and Student’s *t*-test to the following groups: female infected to female control (F inf), male control to female control (MVF CTRL), male infect to female infected (MvF inf) and male infected to male control (M inf). Each color represents a module in the constructed gene co-expression network by WGCNA. The heat map is colored from red (1, positive) to blue (− 1, negative) to indicate the level of correlation of each module with the group of interest. *(*p* < 0.05). ** (*P* < 0.01). ***(*P* < 0.001)

WGCNA was performed in order to identify gene and miRNA interactions and relate gene-miRNA networks to infected fish. The power was set at 14 based on the scale free topology model fit (Supplementary Table S1, and Figure S4A) and mean connectivity (Supplementary Figure S4B). The dendrogram resulting from the WGCNA provides a comprehensive view of the relationships between genes based on their expression patterns (Fig. 4A). The hierarchical clustering depicted in the dendrogram organizes genes into the following modules, named after colors: yellow (519 genes), turquoise (6,294 genes), red (826 genes), blue (2,393 genes), green (8,849 genes) and brown (185 genes) (Fig. 4B). In M inf, module brown and module turquoise were most significantly correlated (correlation value 0.97, *p*-value 2.24E-10 and correlation value 0.71, *p*-value 0.002, respectively). Modules pertaining to inherent sexual dimorphism trait, i.e. significant for both MvF ctrl and MvF inf, were turquoise (correlation value 0.98, *p*-value 2.50E-11 in MvF ctrl and correlation value 1, *p*-value 1.07E-20 in MvF inf) and green (correlation value 0.95, *p*-value 9.64E-09 in MvF ctrl and correlation value 1, *p*-value 6.52E-17 in MvF inf, respectively). Furthermore, sexual dimorphism expression patterns due to infection were identified in three modules: red, brown and blue (significant for MvF inf, *p*-value 7.61E-13, 2.02E-12, 2.68E-05, respectively, with correlation values 0.99, 0.99 and 0.85, respectively). No modules were significantly associated with female infected against female control (F inf).

To further highlight the significant modules related to infection, module membership vs. gene significance was calculated for M inf and MvF inf significant modules MvF inf. M inf for the brown module resulted in a correlation coefficient of 0.72 and *p*-value 7.5E-31 (Supplementary Figure S5A). For MvF inf modules, the blue module resulted in a correlation coefficient of 0.38 and *p*-value 4.6E-83 (Supplementary Figure S5B), the red module resulted in a correlation coefficient of 0.82 and *p*-value < 1E-200 (Supplementary Figure S5C) and the brown module in a correlation coefficient of 0.83 and p-value 2.8E-48 (Supplementary Figure S5D).

### Connections in infected testes

**Fig. 5:**
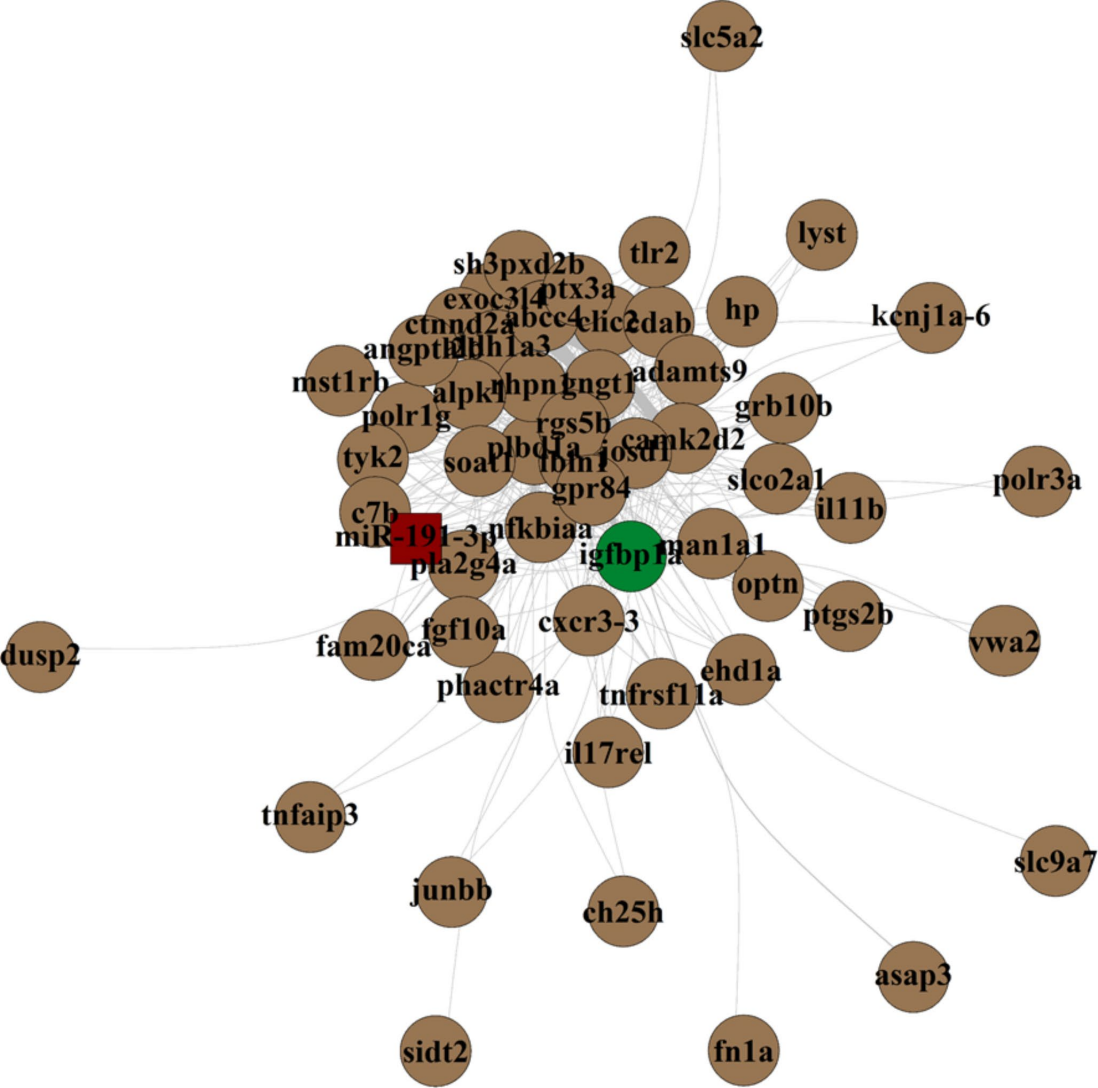
Network analysis in the brown module obtained from Weighted Correlation Network Analysis (WGCNA) to study the correlation between the transcriptome and the miRNonme in the infected testes of European sea bass 48 h after intraperitoneal infected with *Vibrio anguillarum*. In green, insulin-like growth factor binding protein 1a (*igfbp1a*) resulted the hub node. In red, miR-191-3p, identified as being interconnected in the module

With the aim of finding correlation between alteration in the transcriptome and the miRNome in the infected gonads, a network analysis was performed in the brown module in the M inf group, which based on the WGCNA, had the highest significance. After filtering by DEGs of infected male vs. control male, removing unnamed genes and passing a TOM threshold of > 0.3, a total of 54 genes and one miRNA, namely miR-191-3p, remained in the brown module. The hub node of the brown module, being the major player in the module, was insulin like growth factor binding protein 1a (*igfbp1a)* (Fig. 5). Results might indicate that *igfbp1a* was involved in the testicular response of the immune system.

### Sexual dimorphism in the immune response

In order to describe differences between males and females in managing the infection, several strategies were performed. First, DEGs between sexes were identified (Supplementary Figure S6A). To discard the inherent sexual dimorphism, DE between control male and control female was assessed, as well as between infected male and infected female. Then, DEGs between the two control and two infected sexual groups were compared and overlapping genes were discarded from further analysis. In total, 1,347 genes were DE between control males and control females, but not in infected males and infected females. Whereas 2,768 genes were DE between infected males and infected females, but not between the two control groups, making a total of 4,115 unique DEGs (Supplementary Figure S6B). Of these unique DEGs in the control group, 906 DEGs were male-skewed whereas 441 were female-skewed. In infected groups, 1,577 DEGs were male-skewed whereas 1,191 were female-skewed (Dataset 1).

Secondly, to decipher transcriptome and miRNAome immune networks that differ due to the sexual dimorphism, the modules that were significant for MvF inf, but not significant for MvF ctrl (i.e., modules red, blue and brown Fig. 4B) were selected. Significant genes in the modules were filtered based on DEGs and a network with a threshold of > 0.45 was created to visualize the interactions between genes and miRNAs (Supplementary figure S6C). No single gene played a central role in all the connections in the network, but rather was formed by two big clusters, one with neuropeptide FF receptor 2 (*npffr2a*) and sperm autoantigenic protein 17.1 (*spa17.1*) genes, and protein arginine methyltransferase 9 (*prmt9*) gene in the other cluster, all three showing higher connectivity in the network. Notably, no miRNAs passed the threshold.

**Fig. 6:**
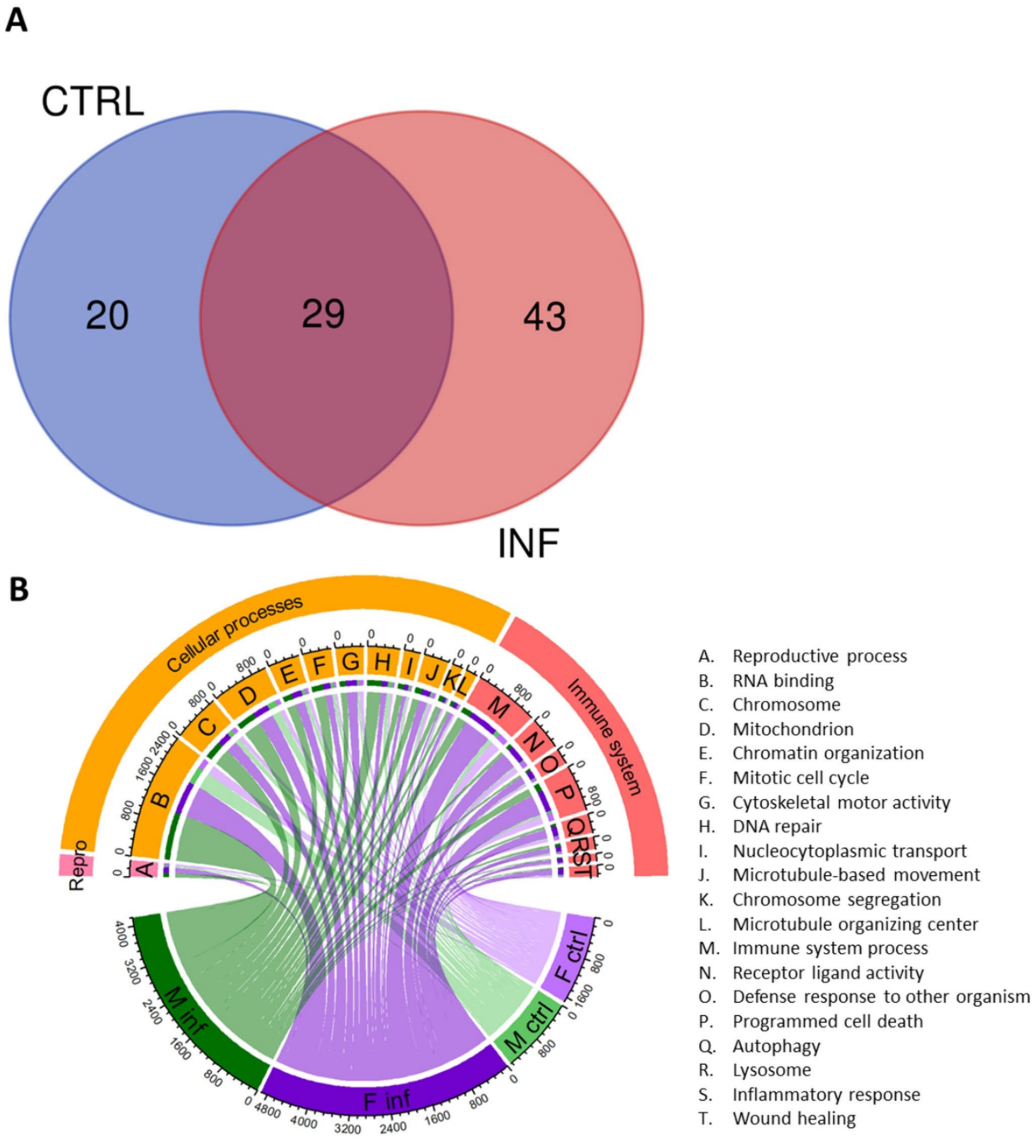
miRNA showing sexual dimorphism. (**A**) Venn diagram of miRNA in both sexes. In blue, differentially expressed miRNAs (DEM) between control males and control females, in red DEM between infected males and infected females. (**B**) Biological functions of the predicted target genes between sexes. M inf: infected males; F inf: infected females; M ctrl: control males, F ctrl: control females. A. reproductive process; B. RNA binding; C. chromosome; D. mitochondrion; E. chromatin organization; F. mitotic cell cycle; G. cytoskeletal motor activity; H. DNA repair; I. nucleocytoplasmic transport; J. microtubule-based movement; K. chromosome segregation; L. microtubule organizing center; M. immune system process; N. receptor ligand activity; O. defense response to other organism; P. programmed cell death; Q. autophagy; R. lysosome; S. inflammatory response; T. wound healing

Additionally, differences in miRNA expression between infected males and females were identified as well as between control males and females (Supplementary Figure S7). DEMs between the two control and the two infected groups between male and females resulted in 49 and 72 miRNAs, respectively (Dataset 4). The overlapping 29 miRNAs shown in the Venn diagram were discarded from further analysis since they were DEMs founds in both sexes regardless of being control or infected (Fig. 6A). Thus, 20 miRNAs were DEM between male control and female control being 12 miRNAs upregulated in females and 8 miRNAs in males. In infected fish, 43 miRNAs were DE being 22 upregulated in infected females and 21 upregulated in infected males.

Target genes of the sex-skewed miRNAs (i.e., 20 for control and 43 for infected, Fig. 6A) were identified following the same criteria as that for identifying the four DEMs in infected males. After filtration, there were 474 and 804 target genes identified in infected males and females, respectively, whereas in control males and females, 133 and 263 target genes were identified, respectively.

Biological functions of the potential target genes were identified by GO slim terms (Fig. 6B). Notably, larger number of potential targets were found in infected females followed by infected males. The highest number of GOs were related to “catalytic activity, signaling, organelle and anatomical structure development (Dataset 7). Interestingly, infected female-skewed miRNAs mostly targeted immune-related transcripts, i.e. 666 genes related to immune system process, 482 to programmed cell death, 375 to receptor ligand activity, 212 to autophagy, 172 to defense response to other organism, 135 related to wound healing, 115 to lysosome and 84 to inflammatory response. On the other hand, miRNAs upregulated in infected males compared to females mostly targeted mitosis and DNA processes-related genes, i.e., 1067 to RNA binding, 435 to cytoskeleton, 327 to DNA repair, 274 to protein catabolic process and 260 to mitotic cell cycle.

## Discussion

The reproduction and the immune system are interconnected and are responsible for the immune defense in the gonads, but also can explain the sexual dimorphism in the immune response. In vertebrates, sex plays a crucial role in shaping the immune response, resulting in differing susceptibilities to diseases between males and females (Nunn et al. 2009; Caballero-Huertas et al. 2024). In particularly in fish, the differing immune responses between sexes have not been fully explored, and their broader implications have often been overlooked. In this study, we wanted to shed light, on (a) on the molecular immune responses after infection in the ovaries and the testes and, (b) on the sexual dimorphism of the immune response in European sea bass, a high valuable cultured fish. By selecting the most resilient sexual phenotype, aquaculture can benefit from various strategies. For example, it can reduce the use of antibiotics and vaccinations, mitigate stress caused by sex-dependent aggressive behaviors by balancing the ratio of males to females, and produce desired sexual phenotypes with greater disease resilience through (epi)genomic selection programs for broodstock. Additionally, understanding the role of sex can help policymakers better interpret disease development, symptoms, and immune system responses.

The present study, demonstrated that testes had a higher alteration of both the miRNAome and the transcriptome after infection. Males altered the testicular genes ~ 26% more than females (2,624 vs. 101 DEGs) and four miRNA were significantly expressed after infection in males, while none were identified in females. Resulting data implied that fish ovaries were more robust in front of infections, indicating a faster clearance of the pathogen. Sexual dimorphism in infections and immune responses has been observed across invertebrates and vertebrates (Nunn et al. 2009). From evolutionary point of view, females are generally less susceptible to infections (Moore and Wilson 2002; Vincze et al. 2022). For example, in *Nephrops norvegicus*, female infection rates with a dinoflagellate parasite peaked during the pre-laying period (Farmer 1974; Stentiford et al. 2020). Similar differences are seen in vertebrates, with higher haemoprotozoan infection prevalence in male pigeons and poultry, while sapelovirus infections in pigs are slightly more common in females (Dey et al. 2010; Kumari et al. 2020). These findings emphasize the importance of pathogen, species, and host-specific factors, as exemplified by Bateman’s principle, where female *Drosophila* prioritize immunity for longevity, while males invest in mating at the expense of defense (Bateman 1948; Rolff et al. 2005). In humans, the study of sexual dimorphic differences in the immune system has found that a large number of immune system genes are located on the sexual chromosome X, which may explain why women present a more active immune system against pathogens. In contrast, women are more sensitive to autoimmune diseases (Mauvais-Jarvis et al. 2020). In fish, several factors determine the sex prevalence in front of a diseases, such as behaviour, social hierarchies, size and lifespan. For example, fish personality shaped the immune system, where aggressive fish have higher expression levels of genes related to innate response (MacKenzie et al. 2009). The prevalence of infection of the parasite *P. neurophilia* was higher in male zebrafish when compared to females, although the vertical transmission was based on oocytes, causing severe problems in zebrafish facilities around the world (Caballero-Huertas et al. 2021). In the current analysis, we observed that upregulated DEGs in the infected males were related to RNA synthesis processes and the immune response while downregulated genes were related to microtubule activity, being “cilium assembly” or “dynein complex” the most enriched, both processes being involved in the sperm motility (zur Lage et al. 2019). These results indicated that the cells in the testes invest the energy to activate the immune system while the sperm-related processes required for reproduction remain inhibited during infection. miRNAs are traditionally understood to suppress the expression of their target genes. However, the mechanism through which miRNAs regulate gene expression is not always straightforward and may involve more complex interactions beyond simple suppression. Furthermore, the potentially low accuracy of target gene predictions in available databases could contribute to inconsistencies in observed regulatory patterns. These factors may help explain the occurrence of upregulated DEGs even when their corresponding miRNAs are also upregulated. To better understand miRNAs that inhibit the expression of their targets, we focused on downregulated DEGs, filtering them to identify those with expression levels inversely correlated with specific miRNAs. This approach aims to clarify the regulatory relationship between miRNAs and their target genes.

Although sexual dimorphism manifests in infections and immune responses across evolutionary history, both in invertebrates and vertebrates (Nunn et al. 2009), scarce data on the molecular mechanisms is available to date. With the goal of discerning sexual dimorphic patterns in fish post-infection, the potential targets of the sex-biased miRNAs exhibiting negative differential expression in either infected males compared to females or control males compared to females, were examined. This mirrors the inhibitory role of the miRNAs in post-transcriptional regulation. Interestingly, this analysis revealed nearly double the number of potential targets regulated in infected females compared to infected males, with a significant portion targeting genes associated with the immune system. These findings suggest that females employed distinct miRNomic and transcriptomic pathways compared to males. Specifically, females appeared to utilize a greater number of miRNAs to inhibit the expression of immune-related genes in response to infection, probably as a mechanism to suppress the immune response after clearing the pathogen. In contrast, as previously described, males seemed to upregulate the immune-related genes in the testes as strategy to cope with the infection in which large number of genes in the transcriptome were involved. Present data underscores the crucial role of sex-specific responses in shaping the host’s defense mechanisms against infections in fish.

Generally, *V. anguillarum* has been described to be highly present in the bloodstream and in the haematopoietic tissue in fish (Frans et al. 2011) but in the fish gonads data is scarce. Here, we detected four animals with presence of *V. anguillarum* in the gonads and two more in the head-kidney. Recent study in European sea bass in Turkish aquaculture farm revealed that *V. gigantis* growth was detected in female gonads of deceased fish, whereas no bacterial growth was found in the male gonads (Yilmaz et al. 2023). In (Schmidt et al. 2017), intraperitoneal injections were executed in zebrafish and *V. anguillarum* presence was measured by immunohistochemistry at multiple time points, i.e. 30 min, 2 h, 8 h and 24 h, in multiple tissues (spleen, liver, swim bladder and peritoneal cavity and blood). Notably, gonads were not tested. In mollusks, *Vibrio* bacterial infection was monitored in male and female gonads. In live animals, higher numbers of bacteria was found in biopsies of mollusk female gonads compared to male gonads, suggesting a greater resistance to infection in male mollusks (Sainz-Hernandez and Maeda-Martinez 2005). Based on the data, we can foresee that recognizing the prevalence of sex in farmed animals is crucial for fisheries management, aquaculture practices, and conservation initiatives.

With the aim of identifying miRNAs as molecular markers of the immune response to bacterial infections in the gonads, we identified four miRNAs altered in the current study; miR-191-3p and miR-183-5p were found to be upregulated whereas miR-451-5p and miR-724-5p were downregulated 48 h after bacterial infection in testes. The miRNA-183/96/182 cluster, of which miR-183 takes part, had a key role in macrophage regulation in mice exposed to *Pseudomonas aeruginosa* and lipopolysaccharide (LPS) by increasing its expression (Muraleedharan et al. 2019) as similarly found in infected testes in European sea bass in the current study. Furthermore, knockdown of the miR-183/96/182 cluster resulted in lower cytokine expression (Ichiyama and Dong 2019; Muraleedharan et al. 2019). In T-helper 17 cells, miR-183 was responsible to increase cytokine production and pathogenicity of Th17 cells (Ichiyama et al. 2016; Wang et al. 2023). miR-191, one of the key nodes in gene regulatory network of infected testes vs. control, was reported to regulate T cell homeostasis, erythroblast enucleation, angiogenesis and T cell survival in mammals (Zhang et al. 2011; Lykken and Li 2016; Gu et al. 2017). Targets of miR-191 include transcription factors, such as SATB1, RIOK3 (RIOkinase 3) and notably, sex gene SOX4 SRY and cell cycle regulators, such as CDK6 (cyclin-dependentkinase 6) and CCND2 (cyclin D2) (reviewed in (Nagpal and Kulshreshtha 2014). Scarce data is found in fish, but a recent experiment in rainbow trout also correlated miR-191 in the immune system as it was altered 48 h after hematopoietic necrosis virus (IHNV) infection in the liver (Wu et al. 2022).

The role of the miR-451 in the immune system has been deeply studied in mammals (Rosenberger et al. 2012; Chapman et al. 2017) (Wang et al. 2015). For many years it is also known that in fish miR-451 plays a crucial role in promoting erythroid maturation (Pase et al. 2009) with the aim of being an informative molecular marker of the immune system. Its expression was lower in bacterial infected in the mucus of the Chinese tongue sole (*Cynoglossus semilaevis*) (Zhao et al. 2021) while being upregulated 48 and 72 h in macrophages immune stimulated by a viral mimic insult in the in Atlantic cod (*Gadus morhua*) (Eslamloo et al. 2018). Its role was also correlated in the early embryonic development in fish subjected to high temperature (Papadaki et al. 2022) and to toxicity (Jenny et al. 2012). Interestingly, miR-451 showed sexual dimorphism in the brain and was related to masculinization process in Atlantic halibut (Bizuayehu et al. 2012). Here, miR-451 was upregulated in the testes in European sea bass together with miR-724-5p. Similarly, miR-724-5p was highly conserved in the fish gonads, showing higher expression in males compared to females in multiple species (van Gelderen et al. 2024), for example in yellowfin seabream (*Acanthopagrus latus*) and Atlantic salmon (Skaftnesmo et al. 2017). More evolutionary-related data indicated that miR-724 is fish-specific miRNA (Li et al. 2014; Zhao et al. 2022), highlighting its potential role as a marker in fish. Besides its role in the reproductive system, miR-724-5p was implicated in immune response pathways although little information is available. For example, miR-724 targeted immune genes in silver carp (*Hypophthalmichthys molitrix*) spleen (Zhao et al. 2024) and in common carp, it was upregulated in spleen due to an inflammatory damage by cadmium (Chen et al., 2021). Our data showed that European sea bass exposed to *V. anguillarum* exhibited downregulation of miR-724-5p in the testis.

Lastly, the network analysis of the crosstalk between microRNome and transcriptome in the infected males gave more information regarding the molecular mechanisms performed in the infected testes. The gene *igfbp*, which belongs to the insulin-like growth factor binding protein family is primarily expressed in the liver, but also it encodes a protein that circulates in the plasma and binds to both insulin-like growth factors (IGFs) I and II (Firth and Baxter 2002; Lay et al. 2021). This characteristic makes, in human, to be a promising biomarker for cancers (Huang et al. 2024). Its role in the reproduction system was characterized in polycystic ovary syndrome in humans (Jin et al. 2023) and in fish, *igfbp* played multiple functions in metabolism, osmoregulation, reproduction, behavior, and immunity (Reindl and Sheridan 2012). In Chinook salmon and Atlantic salmon, circulating higher levels of *igfbp* in plasma were related to stress response (Shimizu and Dickhoff 2017; Breves et al. 2020). Our data manifested that *igfbp* had a relevant role controlling the testicular gene network but also to the post transcriptional control as the miR-192 was enriched in the network analysis. Overall, the data suggest that igfbp1a could serve as a potential marker for the testicular immune response following bacterial infections.

## Conclusions

In summary, our study highlights the intricate relationship between reproduction and the immune system, which not only defines immune defense in gonads but also contributes to sexual dimorphism in the immune response. Current findings revealed that testes exhibited a higher degree of molecular alterations at the microRNome and transcriptome level in response to infection compared to ovaries. Specifically, infected males showed a greater alteration in testicular genes and miRNA expression compared to females. Furthermore, testicular analysis of miRNA expression identified several key molecules involved in immune regulation. Notably, miR-183-5p and miR-191-3p were upregulated, while miR-451-5p and miR-724-5p were downregulated following bacterial infection in testes. In contrast, when we performed the sexual dimorphic study by subtracting only those DEM related to infection and sex, larger number of target genes were inhibited in females which were mostly related to the immune system. The DEGs and miRNAs identified in this study could serve as potential candidates for biomarkers in non-invasive monitoring of fish health. This approach could aid in selecting desired sexual phenotypes that are more resilient to infections. Overall, our study underscores the importance of considering sexual dimorphism in immune responses in fish and provides valuable insights into the molecular mechanisms underlying these differences.

## Electronic supplementary material

Below is the link to the electronic supplementary material.

Supplementary Material 1

Supplementary Material 2

Supplementary Material 3

Supplementary Material 4

Supplementary Material 5

Supplementary Material 6

Supplementary Material 7

Supplementary Material 8

Supplementary Material 9

## Data Availability

Data will be released upon request.
