# Supplementary material for: Gonadal miRNomes and transcriptomes in infected fish reveal sexually dimorphic patterns of the immune response
Source: Funct Integr Genomics. 2025 Jan 30;25(1):29. doi: 10.1007/s10142-025-01537-w (PMC11782434; doi:10.1007/s10142-025-01537-w)
Supplement: Supplementary file 1 — Supplementary Material 1 [file 10142_2025_1537_MOESM1_ESM.docx]

**Supplementary Figures**

**Gonadal miRNomes and transcriptomes in infected fish reveal sexually dimorphic patterns of the immune response**

Tosca A. van Gelderen^1,2^, Pinky Debnath^1,3^, Silvia Joly^1^, Edgar Bertomeu^4^, Neil Duncan^4^, Dolors Furones^4^, Laia Ribas^1^

^1^ Institut de Ciències del Mar, Consejo Superior de Investigaciones Científicas (ICM-CSIC), 08003, Barcelona, Spain

^2^ PhD program in Genetics, Autonomous University of Barcelona, 08193 Bellaterra, Spain.

^3^Department of Fish Biology and Genetics, Sylhet Agricultural University, Sylhet -3100, Bangladesh

^4^Aquaculture Program, Institut de Recerca i Tecnologia Agroalimentaries (IRTA), La Ràpita, Spain

^*^corresponding author: [lribas@icm.csic.es](mailto:lribas@icm.csic.es)


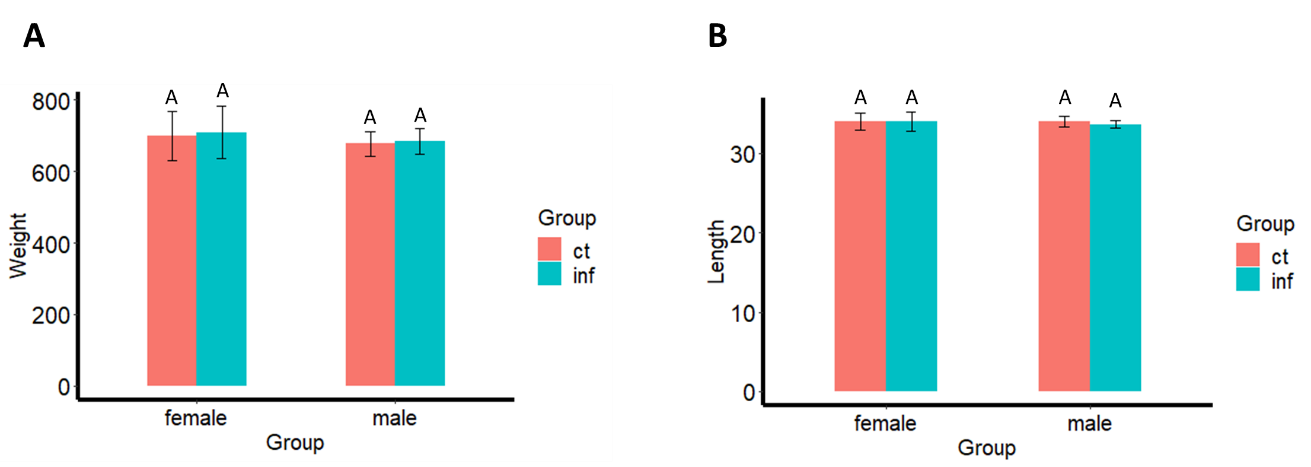


**Supplementary Figure S1**. Supplementary Figure S1: Mean weight (g) and length (cm) of European seabass intraperitoneal infected 48 hours of *Vibrio anguillarum*. Data represents mean ± SD. Similar letters in the bar indicate that data are not statistically significant (*p*<0.05).


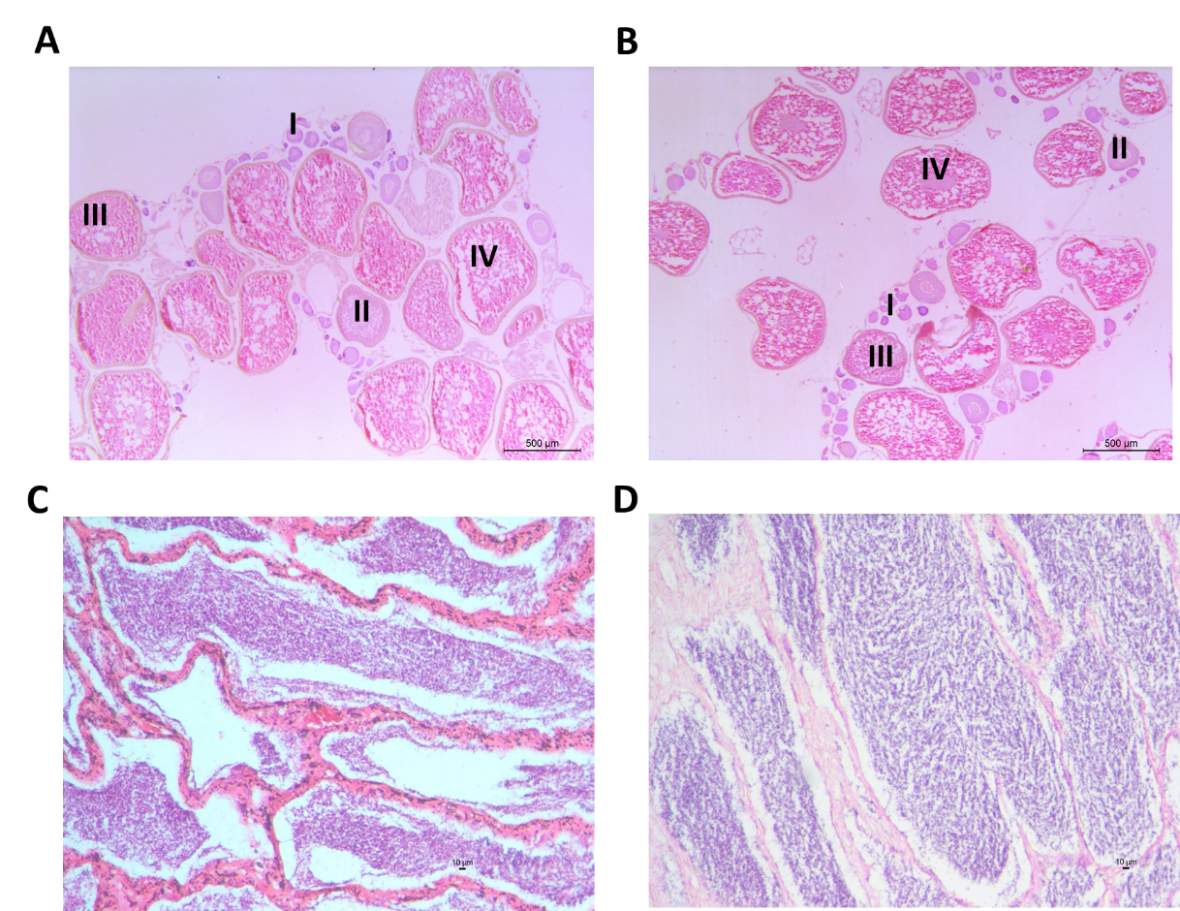


**Supplementary Figure S2**. Histological cross-section of European sea bass gonads in control (A, C) and infected (B, D) ovaries and testes, respectively, 48 h after intraperitoneal infected with *Vibrio anguillarum*. Several stages of oocyte maturation are indicated.


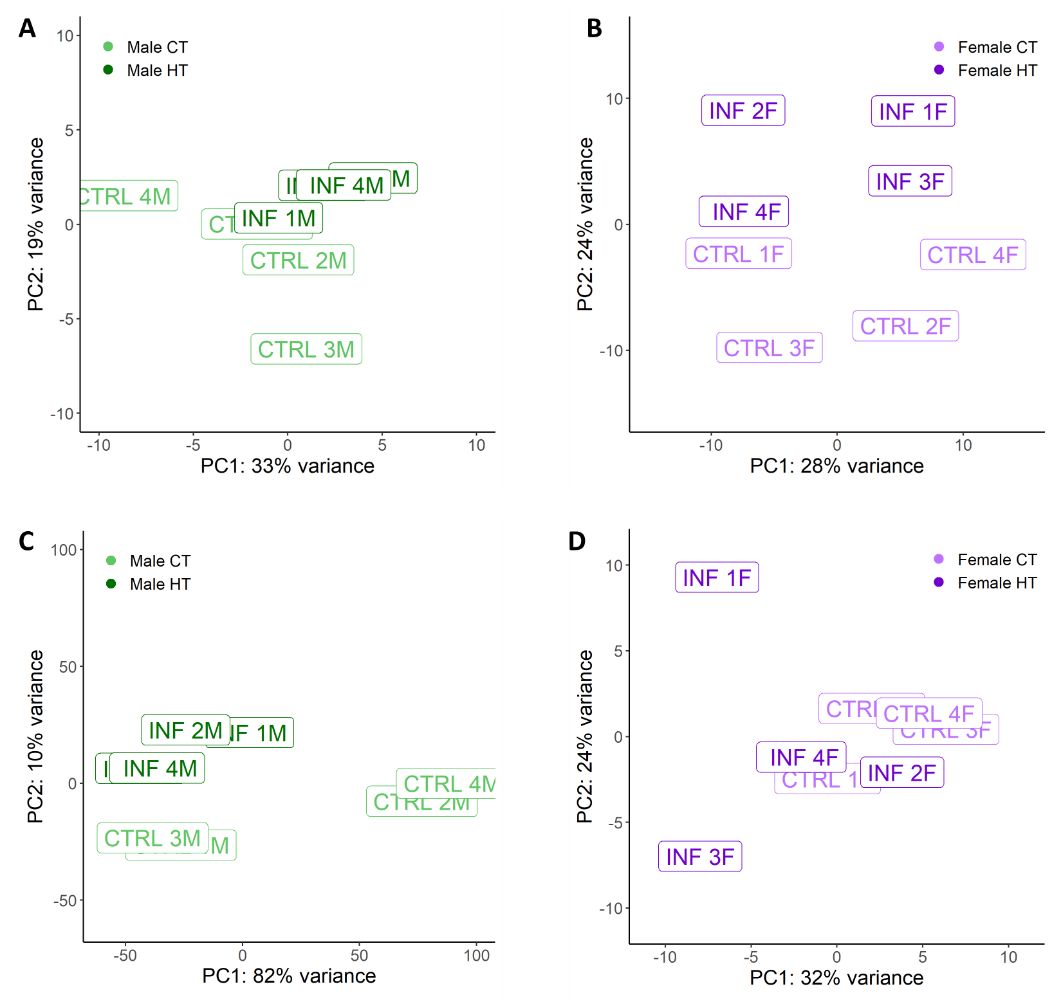


**Supplementary Figure S3**. Principal Component Analyses (PCA) resulting from RNA-sequencing (A, B) and miRNA-sequencing (C, D) in the testes (A, C) and ovaries (B, D), respectively, 48 h after intraperitoneal injection with *Vibrio anguillarum*.


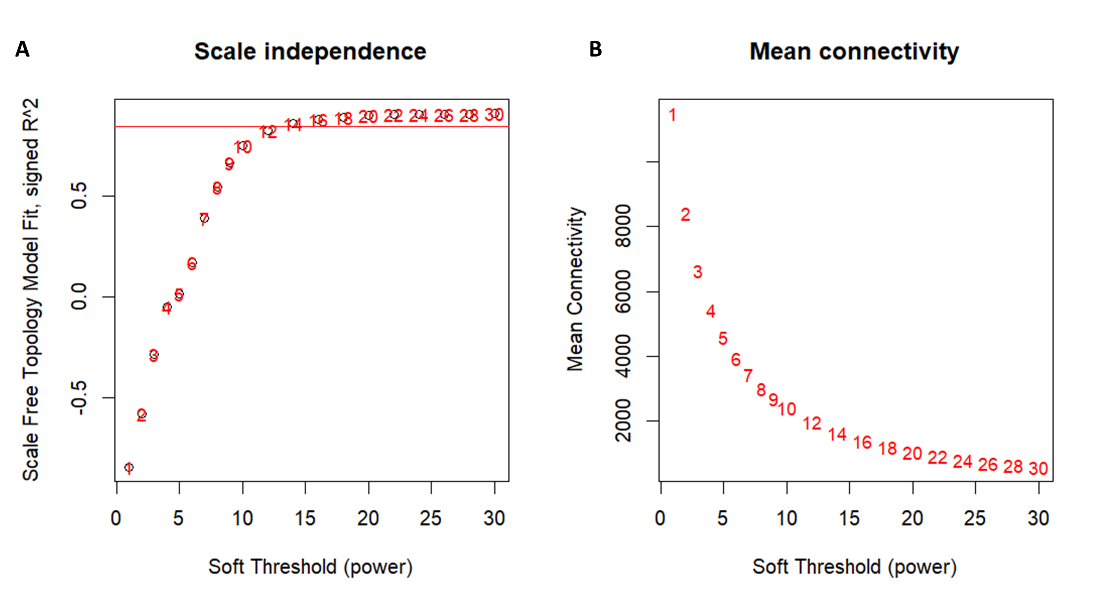


**Supplementary Figure S4**. Scale-independence with threshold R^2^ = 0.85 (A) and mean connectivity (B) of the network in different soft-threshold powers. The left panel displays the correlation of soft threshold with scale-free fit index.


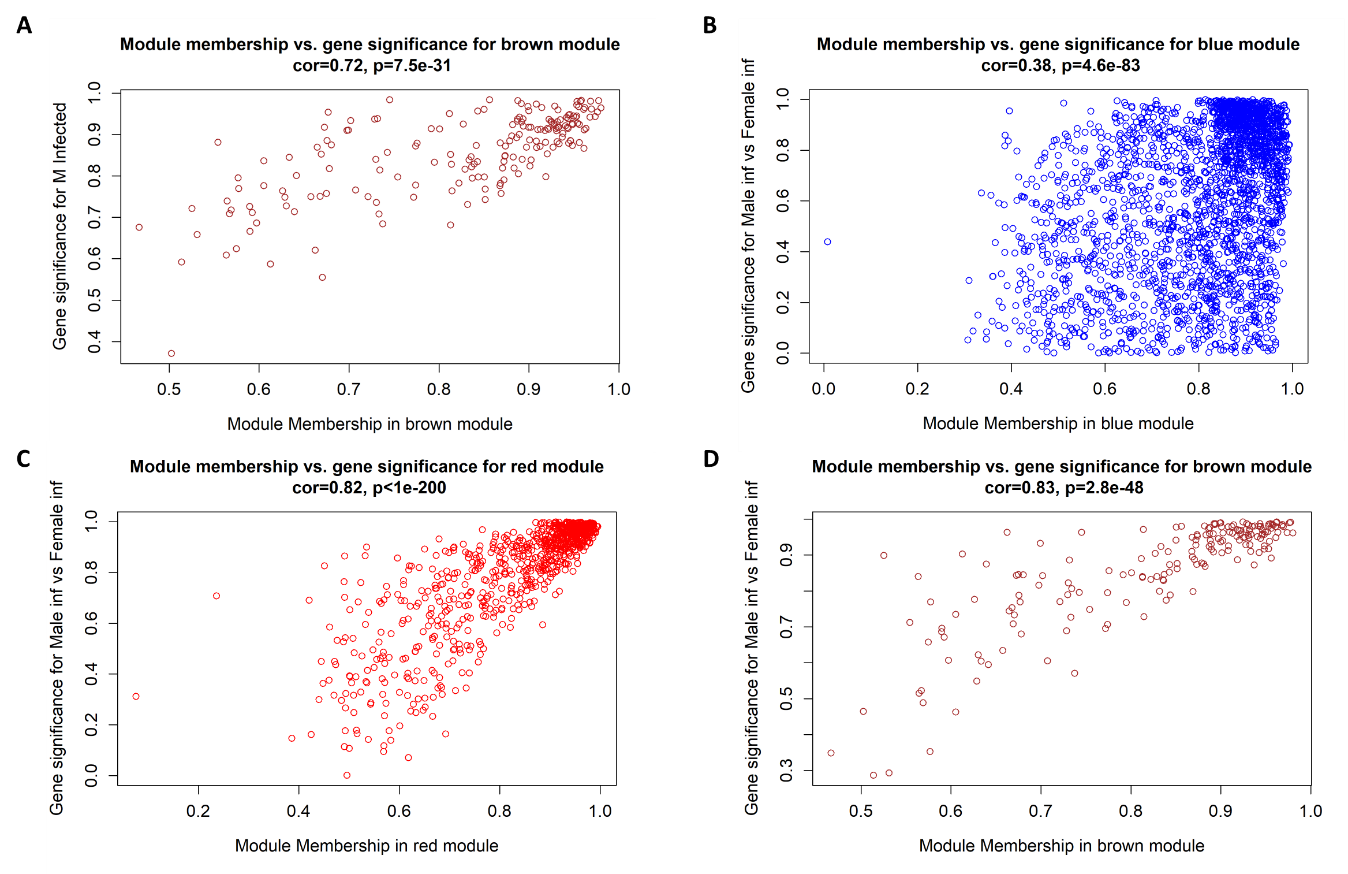


**Supplementary Figure S5**. Scatterplots of correlation between gene significance (GS) versus module membership (MM) of the modules significant for the traits male infected to male control (M inf) and male infected to female infected (MvF inf). Modules were correlated using a Pearson’s correlation and Student’s t-test to the following modules and traits: M inf brown (A), MvF inf blue (B), MvF inf red (C), MvF inf brown (D) and (MvF inf).


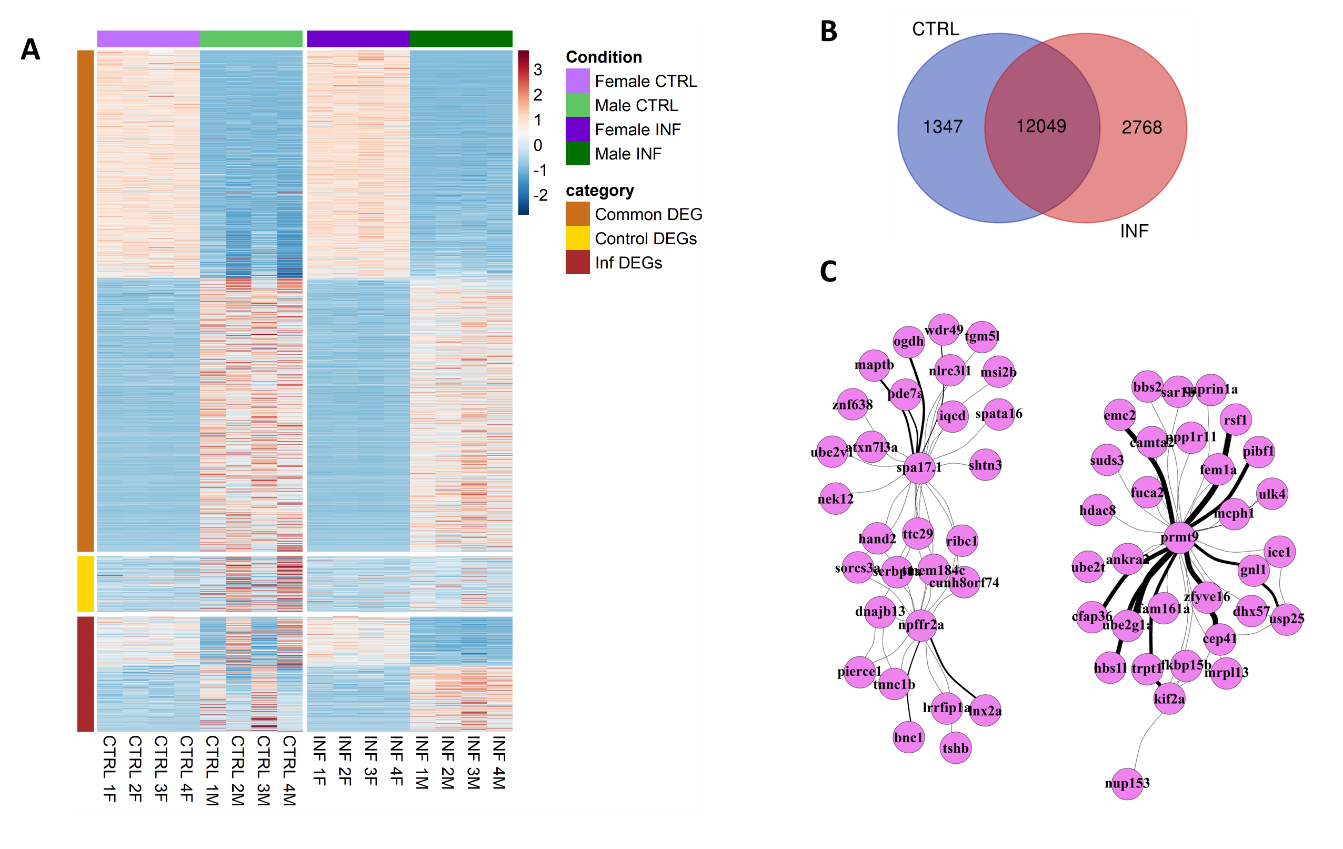


**Supplementary Figure S6**. (A) Heatmap analysis of differential expression genes obtained by RNA-sequencing of the infected testes and ovaries in European sea bass (*Dicentrarchus labrax*). A total of four individuals per group was used. (B) Venn diagram of genes that showed sexual dimorphism. In blue, DEGs between control males and control females, in red, DEGs between infected males and infected females. (C) Network analysis to visualize the interaction between genes responsible for the sexual dimorphism during the immune response in gonads. For the analysis, DEGs of male *vs.* female were used in the red, blue and brown modules from the Weighted Correlation Network Analysis (WGCNA).


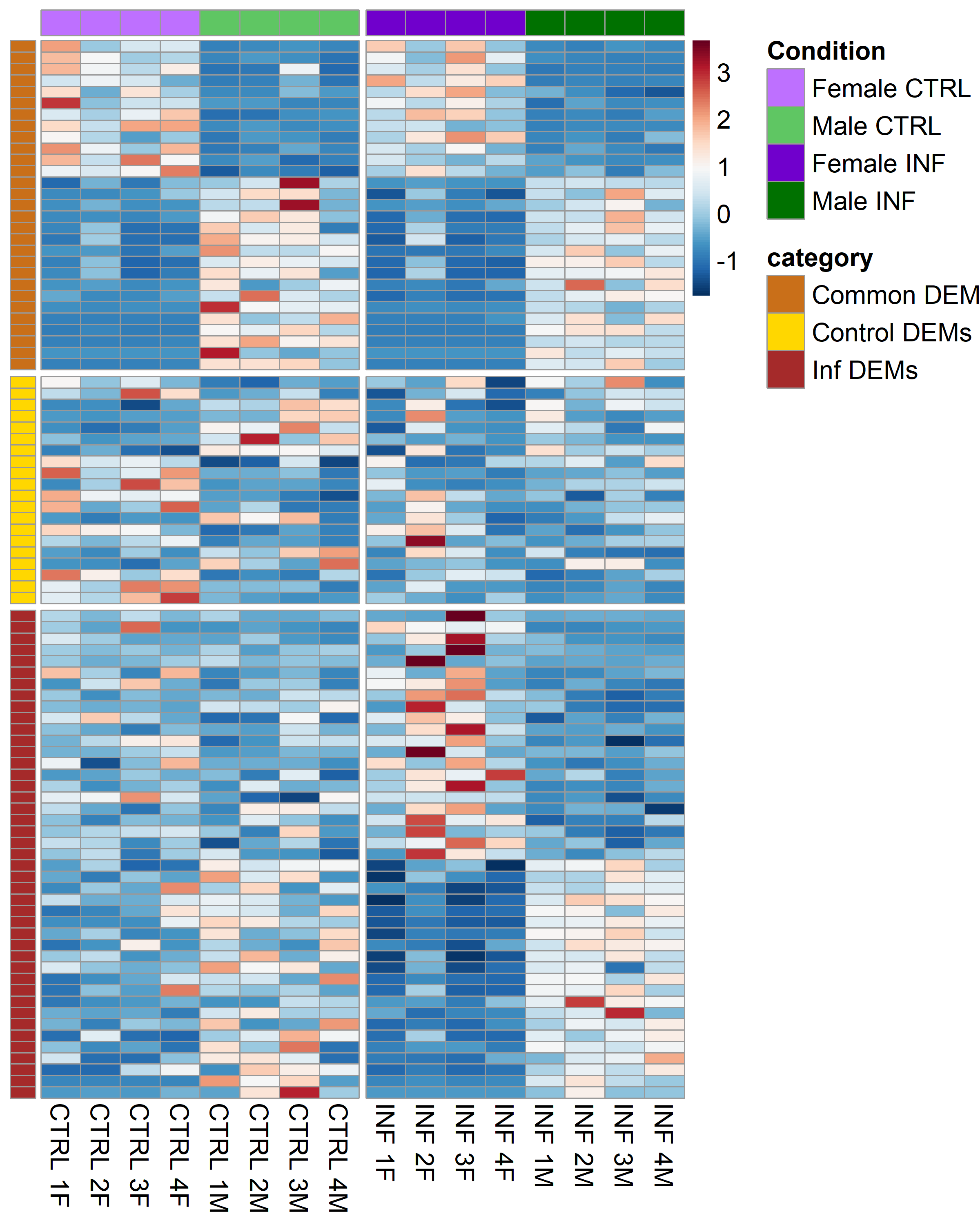


**Supplementary Figure S7**. Heatmap analysis of differential expression miRNAs after sequencing testes (A) and ovaries (B) of European sea bass (*Dicentrarchus labrax*) 48 h after intraperitoneal infected with *Vibrio anguillarum*. A total of four individuals per sex was used.
