# Supplementary material for: Gonadal miRNomes and transcriptomes in infected fish reveal sexually dimorphic patterns of the immune response
Source: Funct Integr Genomics. 2025 Jan 30;25(1):29. doi: 10.1007/s10142-025-01537-w (PMC11782434; doi:10.1007/s10142-025-01537-w)
Supplement: Supplementary file 9 — Supplementary Material 9 [file 10142_2025_1537_MOESM9_ESM.docx]

**Supplementary Table S1**. A list of the most significant miRNAs and predicted-targets genes involved in immune response in each sex.

| **miRNA** | **Sex** | **Group** | **Targets** |
| --- | --- | --- | --- |
| let-7a-3/4/5/6/7-5p | Female | Control | 10 |
| miR-101b-5p |  |  | 17 |
| miR-146a-3p |  |  | 104 |
| miR-181a-5-3p |  |  | 117 |
| miR-183-5p |  |  | 5 |
| miR-214b-5p |  |  | 835 |
| miR-2188-5p |  |  | 1263 |
| miR-222a-5p |  |  | 1088 |
| miR-27a-3p.1 |  |  | 1442 |
| miR-365-2b-5p |  |  | 482 |
| miR-455-5p |  |  | 1152 |
| miR-456-3p |  |  | 617 |
| miR-10b-5p | Male |  | 413 |
| miR-126b-3p |  |  | 2 |
| miR-126b-5p |  |  | 76 |
| miR-138-5p |  |  | 377 |
| miR-145-5p |  |  | 937 |
| miR-218a-5p.1 |  |  | 13 |
| miR-29a-3p.2 |  |  | 16 |
| miR-30c-2-3p |  |  | 277 |
| let-7h-1-5p | Female | Infected | 1524 |
| miR-106b-5p |  |  | 1484 |
| miR-122-5p |  |  | 33 |
| miR-15a-2-5p |  |  | 1049 |
| miR-16a-5p.1 |  |  | 131 |
| miR-16a-5p.2 |  |  | 882 |
| miR-16c-5p |  |  | 128 |
| miR-192-5p |  |  | 0 |
| miR-200b-3p |  |  | 1094 |
| miR-20a-5p |  |  | 1509 |
| miR-210-3p |  |  | 297 |
| miR-212-3p |  |  | 1158 |
| miR-217a-5p |  |  | 1293 |
| miR-222c-5p |  |  | 1090 |
| miR-23a-3p.2 |  |  | 1207 |
| miR-26a-1b-5p.2 |  |  | 1128 |
| miR-30e-1-3p |  |  | 1653 |
| miR-34a-5p |  |  | 318 |
| miR-451-5p |  |  | 26 |
| miR-454b-3p |  |  | 1213 |
| miR-7132b-3p |  |  | 326 |
| miR-93-5p |  |  | 280 |
| dla-nov-2 | Male |  | 122 |
| dla-nov-9 |  |  | 713 |
| let-7a-3/7-3p |  |  | 611 |
| let-7b-3p |  |  | 608 |
| let-7b-5p |  |  | 1045 |
| let-7c-2-5p |  |  | 492 |
| let-7i-1-3p |  |  | 58 |
| miR-125a-1-5p |  |  | 808 |
| miR-125b-5p |  |  | 540 |
| miR-128-3-5p |  |  | 197 |
| miR-132b-5p |  |  | 188 |
| miR-153c-3p |  |  | 632 |
| miR-181a-1/2-3p.1 |  |  | 208 |
| miR-191-3p |  |  | 514 |
| miR-199-5p |  |  | 630 |
| miR-212b-5p |  |  | 356 |
| miR-24a-2-5p |  |  | 344 |
| miR-29b-1a-5p.2 |  |  | 255 |
| miR-29b-2a-5p |  |  | 590 |
| miR-499a-5p |  |  | 616 |
| miR-92a-3p |  |  | 654 |
